# Tunicamycin induced endoplasmic reticulum stress promotes apoptosis of prostate cancer cells by activating mTORC1

**DOI:** 10.18632/oncotarget.19277

**Published:** 2017-07-15

**Authors:** Prasun Guha, Engin Kaptan, Padmaja Gade, Dhananjaya V. Kalvakolanu, Hafiz Ahmed

**Affiliations:** ^1^ Department of Biochemistry and Molecular Biology, University of Maryland School of Medicine and Institute of Marine and Environmental Technology, Baltimore, Maryland, USA; ^2^ Department of Microbiology and Immunology, University of Maryland School of Medicine, Baltimore, Maryland, USA; ^3^ University of Maryland Greenebaum Cancer Center, Baltimore, Maryland, USA; ^4^ Current address: The Solomon H. Snyder Department of Neuroscience, Johns Hopkins University School of Medicine, Baltimore, Maryland, USA; ^5^ Current address: Department of Biology, Istanbul University, Vezneciler, Istanbul, Turkey; ^6^ Current address: GlycoMantra Inc., Baltimore, Maryland, USA

**Keywords:** apoptosis, tunicamycin, ER stress, oxidative burst, metastatic cancer

## Abstract

Studies suggest that tunicamycin may work as a therapeutic drug to cancer cells by inducing stress in the endoplasmic reticulum (ER) through unfolded protein response (UPR) and thereby promoting apoptosis. However, mechanisms of the prolonged activation of the UPR under sustained ER stress in the regulation of cell apoptosis are largely unknown. To delineate the role of candidate genes in the apoptotic process under ER stress and to search for new therapeutic strategies to treat metastatic castration resistant prostate cancer, we performed whole genome expression microarray analysis in tunicamycin treated metastatic androgen-insensitive prostate cancer cells, PC-3. Among several induced genes, the expression of eNOS (*NOS3*) gene was remarkably high. The increased expression of eNOS activates mTORC1 through RagC. This results into an accumulation of p62 (SQSTM1) which facilitates aggregation of ubiquitinated protein thus compromising clearance of misfolded toxic protein aggregates. Lastly, association of p62 proteins and misfolded proteins promote reactive oxygen species (ROS) mediated mitochondrial apoptosis. Overall, our data demonstrate that tunicamycin induced ER stress promotes prostate cancer cell death by activating mTORC1 through eNOS-RagC pathway.

## INTRODUCTION

Within the endoplasmic reticulum (ER), nascent proteins are appropriately folded through the actions of a series of molecular chaperones, lectins, and glycosidases and travelled to the Golgi apparatus for further trafficking [1]. When this process is incomplete or if there is an imbalance between the cellular demand for protein synthesis and the capacity of the ER in promoting protein maturation and transport, the cell must deal with any proteins that remain unfolded or misfolded within the ER - the characteristic of the cell is known as ER stress [2, 3]. It is believed that ER stress contributes to wide range of pathogenesis such as liver cirrhosis, diabetes and Alzheimer’s disease [4, 5]. To help alleviate ER stress, controlled transcriptional response, called “Unfolded protein response (UPR)” composed of molecular chaperones promote the correct folding of nascent proteins in the ER and inhibit the aggregation of aberrant proteins. The key component of the UPR, glucose regulated protein 78 (GRP78), dissociates from three types of ER-localized transmembrane signal transducers to lead to their activation [6, 7]. These transducers include two protein kinases IRE1 (inositol requiring kinase 1), and PERK (double stranded RNA-activated protein kinase-like ER kinase) and the transcription factor ATF6 (activating transcription factor 6) [3, 6, 7]. Together they promote correct folding, eliminate faulty protein, and restore ER homeostasis generally by decreasing translation initiation (mediated by IRE1α and ATF6), and selectively inhibiting translation of specific mRNAs (mediated by PERK) [3].

A recent study has shown that cells under ER stress activate autophagy (macroautophagy) [8, 9]. Macroautophagy induced by stress involves the sequestration of cellular organelles by double-layered membranes called autophagosomes, which ultimately fuse with lysosomes and their contents are degraded by lysosomal hydrolases [10]. Autophagy is generally characterized by the presence of cytoplasmic vacuoles and autophagosomes, an increase in cleavage of LC3 (microtubule-associated protein 1 light chain 3), and a reduction in p62 (also known as SQSTM1) protein levels [11]. Under basal autophagy, long-lived proteins and damaged organelles are removed and the degradation products are released into the cytosol as intermediate metabolites.

Like the UPR, autophagy is associated with both cell survival and cell death depending on the level and duration of autophagy [12]. For example, if the stress on ER remains unresolved or in the presence of sustained ER stress, prolonged activation of the UPR leads to mitochondrial apoptosis [13, 14]. The pro-apoptotic Bcl-2 family members Bax, Bak [14], PUMA as well as the transcriptional induction or posttranslational activation of Bcl-2 homology domain 3 (BH3)-only proteins [15] are involved in this mitochondrial apoptosis.

Tunicamycin (Tun), a naturally occurring antibiotic, induces ER stress in cells by inhibiting the first step in the biosynthesis of N-linked glycans in the proteins resulting many misfolded proteins [16]. A few studies suggest that tunicamycin may work as a therapeutic drug to cancer cells as it has been shown to sensitize human colon and prostate cancer cells to TRAIL-induced apoptosis [17, 18]. However, a potential role of the prolonged UPR activation (under sustained ER stress) in the regulation of cell apoptosis is largely unknown. On whole genome expression microarray of Tun-treated PC-3, we discovered eNOS as the most upregulated gene. Under sustained ER-stress, up-regulation of eNOS activated mTORC1 through eNOS-RagC pathway and promoted intracellular accumulation of p62 protein, which in turn, triggered reactive oxygen species (ROS) dependent mitochondrial apoptosis.

## RESULTS

### Treatment of PC-3 cells with tunicamycin (Tun) decreased N-glycosylation production with an induction of ER stress

**Figure 1:**
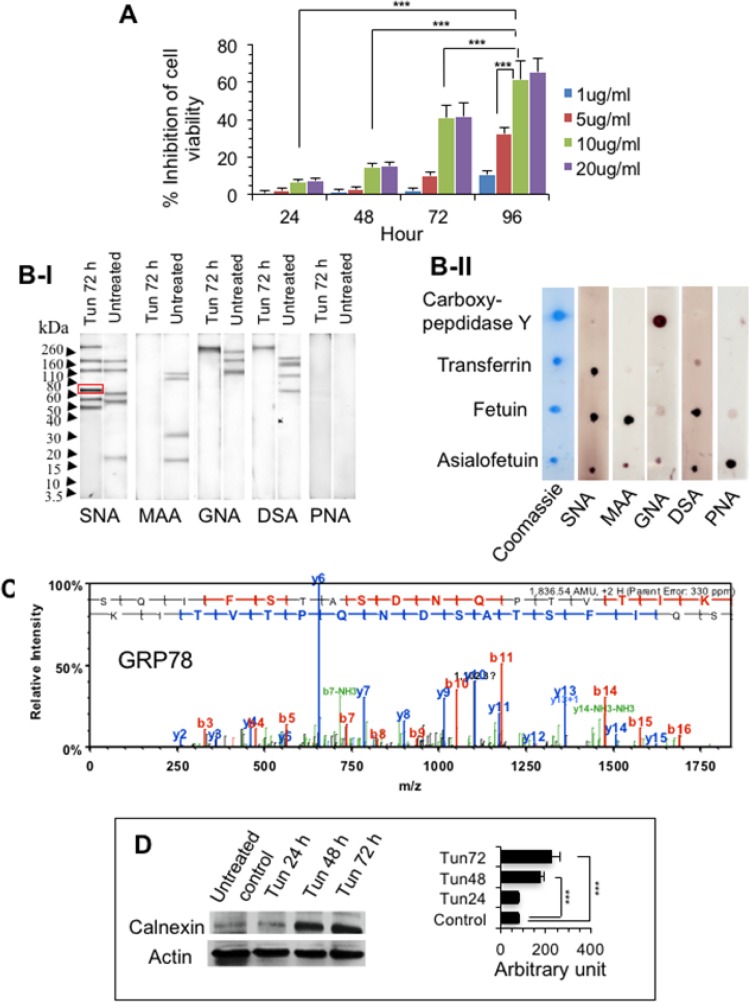
Tunicamycin-treated PC-3 cells show decreased N-glycosylation with an induction of ER stress **(A)** Cell viability of PC-3 cells. 1x 10^4^ Cells were treated with 1-20 μg/ml of tunicamycin (Tun) for 24-96 h and cell viability was assessed with WST-1 staining. **(B)** Glycan differentiation analysis of (I) PC-3 cell extract and (II) standard glycoproteins. PC-3 cells were treated with 10 μg/ml Tun for 72 h and the presence of N- and O-glycosylations in the cell extract and in the standard glycoprotein (as controls) were analyzed by binding with various lectins as described in Materials and Methods. 70 kDa protein band (marked by red rectangle) was subjected to mass-spectrometric analysis. **(C)** Mass spectrum of GRP78. 70 kDa protein (in Figure B-I) was identified as GRP78. **(D)** Representative Western blot showing expression of calnexin. PC-3 cells were treated with 10 μg/ml Tun for 24-72 h and expression of calnexin was determined on W. blot using anti-calnexin antibody. Actin was used as a loading control. The bar diagram at right shows quantification of the calnexin expression from three experiments as measured by Image J software.

To address gene regulation in apoptosis, PC-3 cells were treated with various doses of tunicamycin (1-20 μg/ml) for various time durations (up to 96 h) and cell viability was recorded. Reduction of cell viability was observed overtime up to 96 h and dose-dependently for concentration 1-10 μg/ml of Tun with cell death upto 61.5% (Figure 1A). Untreated control cells were 100% viable (data not shown). As 10 μg/ml Tun treatment of PC-3 cells for 72 h reduced cell viability to ∼41%, which was close to IC_50_, we used that conditions for most follow up cell death experiments.

Because Tun blocks the formation of *N*-glycosylation in the protein, glycan analysis of the Tun-treated and untreated samples was performed with specific lectin binding along with standard proteins using glycan differentiation kit (Roche). Compared to the untreated sample, Tun (10 μg/ml, 72 h) largely suppressed N-glycosylation as demonstrated by binding to *N*-glycan specific lectins such as DSA (*Datura stramonium* agglutinin specific to galactose β1,4 linked to *N*-acetylglucosamine in complex and hybrid *N*-glycans), GNA (*Galanthus nivalis* agglutinin specific to terminal mannose of high mannose *N*-glycans), and MAA (*Maackia amurensis* agglutinin specific to sialic acid α2,3 linked to galactose in *N*- and *O*-glycans) (Figure 1B–I). However, SNA (*Sambucus nigra* agglutinin specific to sialic acid α2,6 linked to galactose in *N*- and *O*-glycans) identified a few extra bands (around 50 kDa and 70 kDa, and above 260 kDa) in the Tun-treated cells compared to the untreated cells, indicating possible presence of O-glycosylated proteins (Figure 1B–II). We performed mass-spectrometric analysis of the 70 kDa band and it turned out to be GRP78 (Figure 1C), a well-known ER stress marker [16, 19]. The induction of ER stress was further confirmed by calnexin [6], whose expression was high at 48 and 72 h compared to untreated control (Figure 1D).

### Sustained ER-stress induced cell death in PC-3 cells

**Figure 2:**
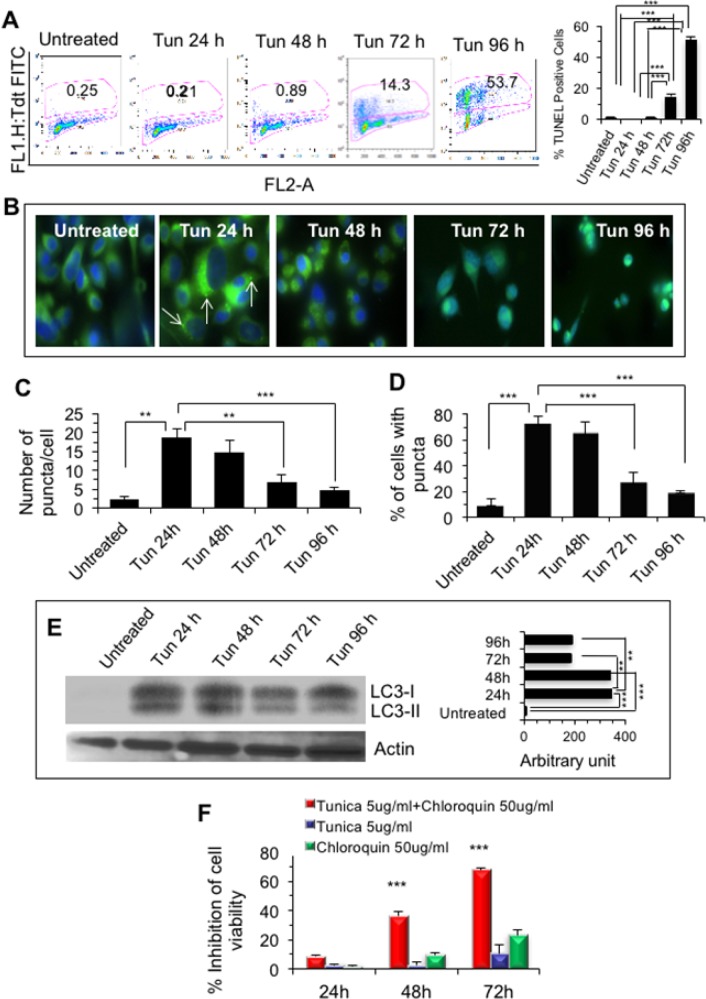
Sustained ER stress induced cell death in PC-3 cells **(A)** TUNEL assay. Cells were treated with 10 μg/ml of tunicamycin for 24-96 h and TUNEL assay was performed on a flow cytometer. *Right.* Bar diagram showing quantitation of TUNEL positive cells represented as ± SD. *** represents as p≤0.001. **(B)** Fluorescence microscopy showing autophagic puncta in LC3-GFP transfected PC-3 cells that were treated with Tun. In 24 Tun treated cells, white arrows represent autophagic puncta. **(C)** Bar diagram showing number of puncta per cell as described in Figure 2B. **(D)** Bar diagram showing number of PC-3 cells with puncta as described in Figure 2B. For C and D, cells were counted under in each field and 5 different fields were scored for statistical analysis. Number of puncta per cell was counted in each field. **(E)** Representative Western blot of Tun- treated PC-3 showing LC3-II (autophagy marker). Approximately 10^6^ cells were applied on SDS-PAGE and subjected to W. blot probed with anti-rabbit MAP1 LC3 antibody followed by incubation with goat anti-rabbit IgG-HRP and development with ECL substrate. Actin was used as a loading control. The bar diagram at right shows quantification of LC3-II from three experiments as measured by Image J software. **(F)** Synergistic cell death of PC-3 cells in the presence of chloroquine and tunicamycin. PC-3 cells were treated with either Tun (5 μg/ml) or chloroquine 50 μg/ml or in combination for 24-72 h and cell death was measured by WST-1 staining.

To examine if Tun-treated cells undergo apoptosis, TUNEL assay was performed. Although no or negligible (<1%) apoptosis was observed during the first 48 h, apoptosis was evident at 72 h (14.3%, p<0.001) and 96 h (53.7%, p<0.001) (Figure 2A). To investigate if induction of autophagy was the reason for cell survival in the initial phase of Tun-treatment, we examined the formation of autophagic puncta after transfecting cells with LC3-GFP. Intriguingly, autophagy was significant at a very early stage of ER stress and persisted after 24 h of Tun treatment as emphasized by increased number of puncta (Figure 2B–2D). However, at later points number of puncta was reduced significantly (Figure 2B–2D). This observation was further validated by Western blot analysis where increased expression of LC3-II (a cleaved and lipidated form of LC3) was evident after 24 h of Tun treatment (Figure 2E). The intensity of LC3-II level remained elevated for up to 48 h, but decreased at 72 and 96 h (Figure 2E). Taken together, cells undergo apoptosis through initial activation of autophagy during sustained ER stress. To assess the importance of initial autophagy activation during ER stress, PC-3 cells were treated with chloroquine in the presence of tunicamycin. Chloroquine raises the pH and reduces autophagic degradation by blocking fusion of autophagic vacuoles with lysosomes. As shown in Figure 2F, synergistic cell death of PC-3 occurred when treated with chloroquine and tunicamycin together suggesting that the initial autophagic activation was important for cell survival.

### Tunicamycin-induced cell death of PC-3 cells was ROS-dependent

**Figure 3:**
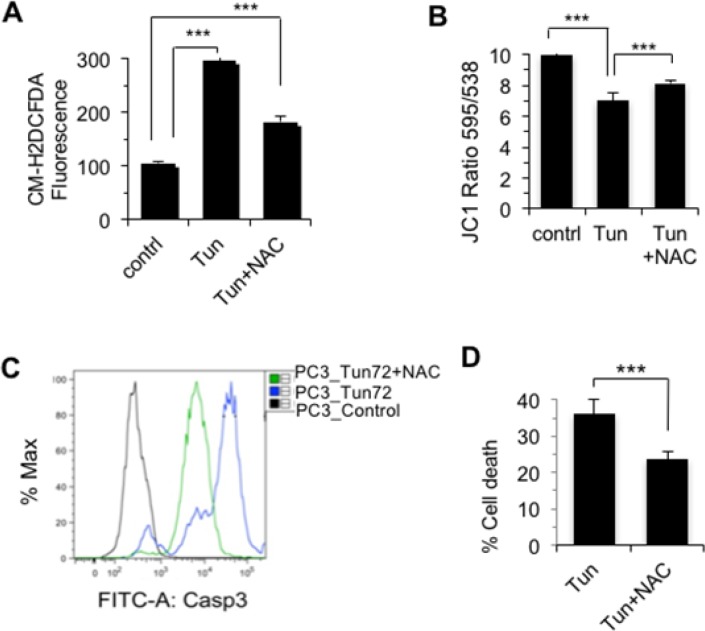
Tunicamycin-induced cell death of PC-3 cells was ROS-dependent **(A)** Effect of Tun on ROS generation. PC-3 cells were treated with Tun (10 μg/ml, 72 h) in the presence or absence of 2.5 mM N-acetyl cysteine (NAC) and ROS was measured with CM-H2DCFDA. **(B)** Effect of ROS in mitochondrial membrane potential. PC-3 cells were treated with Tun (10 μg/ml, 72 h) in the presence or absence of 2.5 mM NAC and membrane potential was measured. **(C, D)** Effect of ROS on cell death. PC-3 cells were treated with Tun (10 μg/ml, 72 h) in the presence or absence of 2.5 mM NAC and cell death was measured by either cleaved caspase-3 staining on a flow cytometer (C) or WST-1 staining (D).

To determine if tunicamycin induced cell death of PC-3 is through reactive oxygen species (ROS) [20], we measured ROS spectrofluorimetrically using ROS detection kit. Compared to the untreated control cells, Tun-treated (10 μg/ml, 72 h) cells showed almost 3-fold accumulation of ROS, which was markedly reduced in the presence of antioxidant N-acetyl cysteine (NAC) (Figure 3A). To explore the impact of ROS, cells were treated with Tun alone or Tun+NAC and analyzed mitochondrial membrane potential and cell death. Tun induced loss of membrane potential, but NAC treatment reduced Tun-mediated loss of dissipation of mitochondrial membrane potential (Figure 3B). NAC treatment also reduced Tun-mediated Caspase 3 activation (Figure 3C) and cell death (Figure 3D). Taken together, data suggest that sustained accumulation of ROS destabilized mitochondrial membrane potential and triggered mitochondrion-dependent apoptosis. However, ROS-independent cell death cannot be ruled out as NAC treatment did not abrogate Tun-induced cell death completely.

### Genome-wide expression analysis identifies important candidate genes for cell death

**Figure 4:**
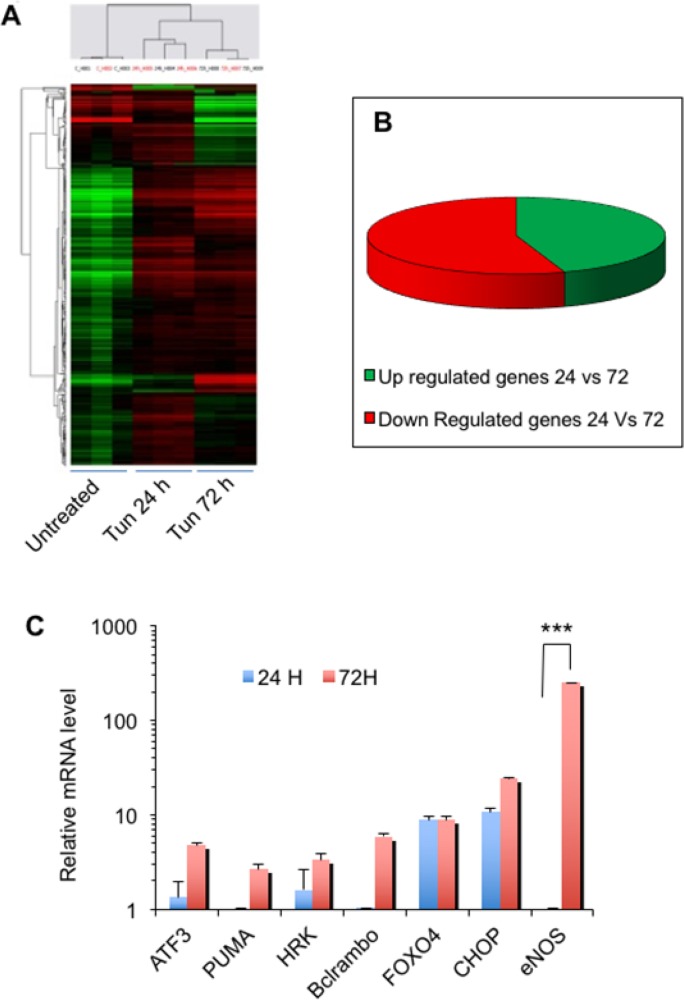
Genome-wide expression analysis identifies important candidate genes for cell death **(A)** Heat maps showing whole genome expression microarray results in triplicate for untreated and Tun treated (24 h and 72 h) PC-3 cells. **(B)** Pie chart showing global gene expression level (green, up-regulation; red, down-regulation). **(C)** Validation of expression of selected genes through quantitative RT-PCR. Expression of each mRNA relative to GAPDH was calculated according to the comparative ΔΔC(t) method. *** represents as p≤0.001.

To investigate gene expression changes associated with apoptosis under sustained ER stress, we chose two time points (24h and 72h) of Tun treatment (10 μg/ml) and performed whole genome expression analyses using microarrays. Of two time points (24 h and 72 h), the former one represents mostly autophagic activation and the latter one indicates apoptosis initiation (please see Figure 2). Microarray results have been deposited to GEOarchive (www.ncbi.nlm.nih.gov/geo) (Accession No. GSE38643) and heat maps are shown in Figure 4A. Microarray data on the 72 h Tun-treated (apoptotic stage) cells were compared with those of the 24 h Tun-treated (no-apoptosis stage) and untreated cells. A total of 653 genes were found up-regulated while 806 genes were down-regulated when 72 h Tun-treated cells were compared with the 24 h Tun-treated cells (Figure 4B). Among the upregulated genes certain pro-apoptotic gene products (such as HRK, Bcl-rambo [BCL2L13], PUMA) and stress-associated transcription factors (e.g. FOXO4, ATF3, CHOP) were induced at 72 h Tun-treatment compared to 24 h Tun-treatment (Microarray data, Accession No. GSE38643). Among all, the eNOS (*NOS3*) gene was most markedly induced. Selected genes were further validated by qPCR analysis (see Table 1 for primer sequences), where eNOS showed significantly increased expression at 72 h compared to 24 h Tun-treated (250-fold, p value <0.001) or untreated cells. (Figure 4C). Microarray and qPCR analyses also showed that change in eNOS gene expression was minimal at 24 h compared to the untreated control.

**Table 1:** Primer sequences used in quantitative RT-PCR

Gene name	Forward primer	Reverse primer
NOS3	5’-ACCCTCACCGCTACAACATC-3’	5’-GAAAACAGGAGTGAGGCTGC-3’
ATF3	5’-GCCATTGGAGAGCTGTCTTC-3’	5’-GGGCCATCTGGAACATAAGA-3’
CHOP	5’-CAGAACCAGCAGAGGTCACA-3’	5’-AGCTGTGCCACTTTCCTTTC-3’
FOXO4	5’-CTTTGAGCCAGATCCCTGAG-3’	5’-TTCCAACAGCATTGCTCATC-3’
HRK	5’-AGGTTGGTGAAAACCCTGTG-3’	5’-CACTTCCTTCTCGAAGTGCC-3’
Bcl-rambo	5’-CGTGGAGAAAGAAGAGGTGC-3’	5’-AGAGATGTAGCAGGGCTGGA-3’
Bcl-xl	5′-TCCTTGTCTACGCTTTCCACG-3′	5′-GGTCGCATTGTGGCCTTT-3
PUMA	5-GAAGAGCAAATGAGCCAAACG-3	5-GGAGCAACCGGCAAACG-3

### eNOS inhibits autophagic degradation and accumulates p62

**Figure 5:**
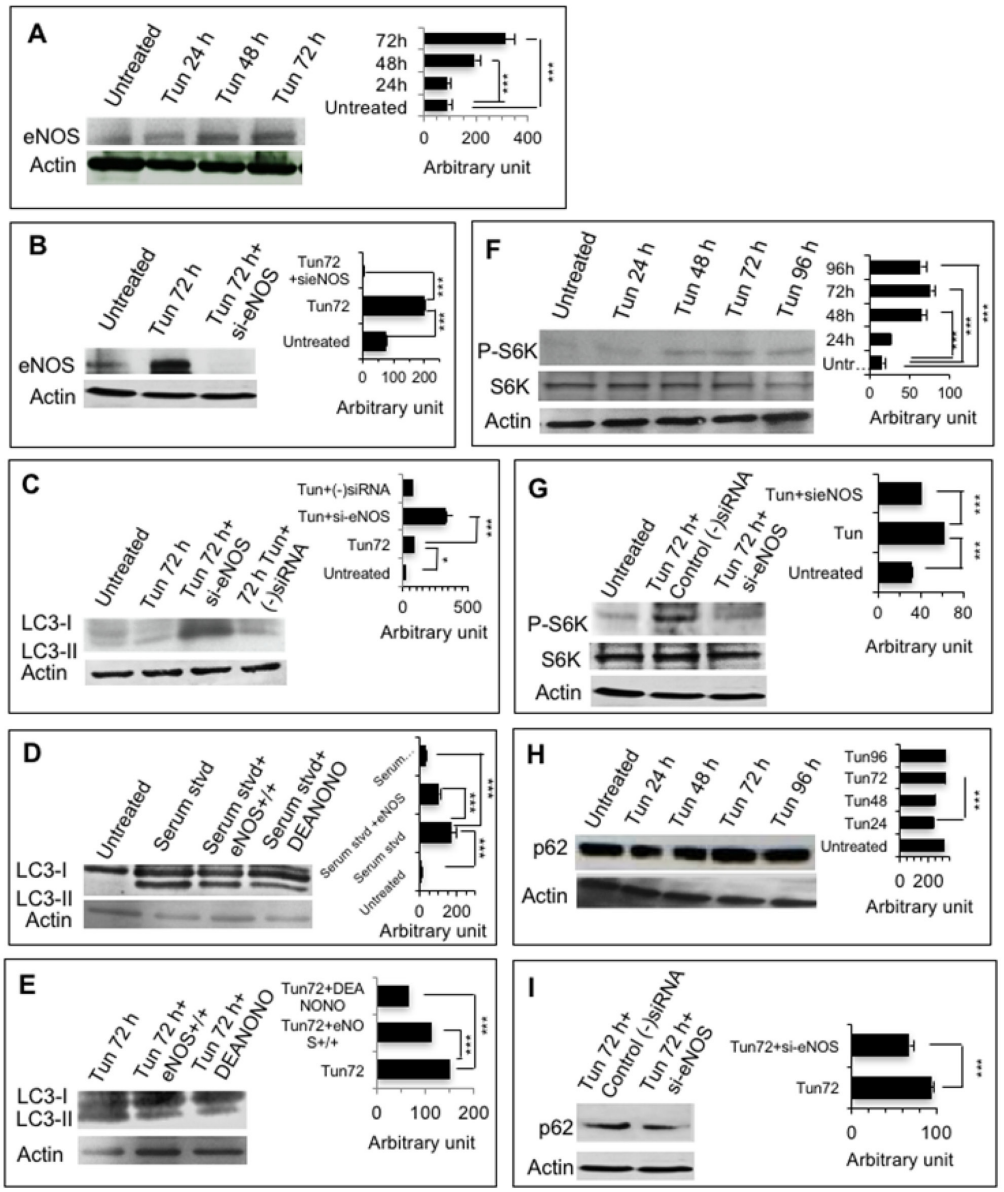
eNOS inhibits autophagic degradation and accumulates p62 **(A, B)** Representative Western blots showing expression of eNOS in PC-3 cells. Cells were treated with 10 μg/ml Tun for 24-72 h and expression of eNOS was determined on W. blot using anti-eNOS antibody. In case of B, cells were treated with siRNA specific for eNOS (si-eNOS) or negative control siRNA for 24 h followed by Tun treatment (10 μg/ml, 72 h). The bar diagrams at right show quantification of eNOS from three different experiments as measured by Image J software. **(C-E)** Representative Western blots showing LC3-II levels in PC-3 cells at various conditions. In case of C and E, cells were treated with si-eNOS or negative control siRNA or eNOS construct (for ectopic expression) or empty vector (for negative control) for 24 h prior to Tun treatment (10 μg/ml, 72 h). In case of D, cells were transfected with eNOS construct (for ectopic expression) or empty vector (for negative control) followed by serum starvation in the presence or absence of NO donor DETA-NONOate (DEANONO) for 6 h. The bar diagrams at right in each case show quantification of LC3-II from three different experiments as measured by Image J software. **(F, G)** Representative Western blots showing expression of S6 kinase (S6K) and phosphorylated S6 kinase (P-S6K) in PC-3 cells. Cells were treated with 10 μg/ml Tun for 24-96 h and expression of S6K and P-S6K was determined on W. blot using anti-S6K or anti-P-S6K antibodies, respectively. In case of G, cells were treated with si-eNOS or negative control siRNA for 24 h prior to Tun treatment (10 μg/ml, 72 h). The bar diagrams at right show quantification of P-S6K from three different experiments as measured by Image J software. **(H, I)** Representative Western blots showing expression of p62 in PC-3 cells. Cells were treated with 10 μg/ml Tun for 24-96 h and expression of p62 was determined on W. blot using anti-p62 antibody. In case of I, cells were treated with si-eNOS or (−)siRNA for 24 h prior to Tun treatment (10 μg/ml, 72). The bar diagrams at right show quantification of p62 from three different experiments as measured by Image J software. In all cases, actin was used as loading control.

Relationship of increased expression of eNOS and activation of mTORC1 was examined using RNAi approach. Expression of eNOS protein in the Tun-treated and untreated PC-3 cells was first analyzed through Western blot. Consistent with microarray data and qPCR analysis, Western blot of eNOS protein expression revealed its marked up-regulation at 72 h Tun treatment (Figure 5A). The ability of si-eNOS to downregulate eNOS expression was then confirmed (Figure 5B).

As eNOS and NO donors were previously shown to inhibit autophagic flux [21], we confirmed the effects of eNOS in our system. To determine if eNOS could regulate autophagic process, we measured LC3-II level after knocking down or overexpressing eNOS. Knockdown of eNOS at 72 h Tun-treated cells stimulated endogenous LC3-II levels compared to Tun alone (Figure 5C). As silencing of eNOS induced autophagy, the above experiment suggested that upregulation of eNOS expression at 72 h of Tun treatment could inhibit autophagic response. To confirm the universal effect of eNOS/nitric oxide on autophagy, the PC3 cells were serum starved to induce autophagy. Ectopic expression of eNOS or presence of nitric oxide donor DEANONO inhibited autophagy (Figure 5D). Furthermore, over expression of eNOS or presence of nitric oxide donor DETA-NONOate also inhibited Tun induced autophagy at 72 h as demonstrated by LC3-II protein levels (Figure 5E). Thus, eNOS negatively modulated autophagy and stalled it at 72 h of Tun treatment.

Activity of mTORC1, a protein kinase, was assessed by the phosphorylation status of its substrate, ribosomal S6 kinase 1 (S6K), at T^389^ [22]. Phosphorylation of S6K at T^389^ increased at 48 h and 72 h of Tun treatment, but became plateau at 96 h Tun treatment (Figure 5F). However, knockdown of eNOS prevented phosphorylation of S6K (Figure 5G). Thus, eNOS could activate mTORC1.

As mTOR negatively modulates autophagy, which results into p62 accumulation [23], we hypothesize that increased expression of eNOS promotes p62 accumulation. In our experiments, we found a marked accumulation of p62 at 72 h compared to 24 h (Figure 5H), but knockdown of eNOS reduced accumulation of p62 (Figure 5I). Thus, under sustained ER stress, upregulation of eNOS activates mTORC1 and promotes accumulation of p62.

### eNOS activates mTORC1 through RagC to inhibit autophagic degradation

**Figure 6:**
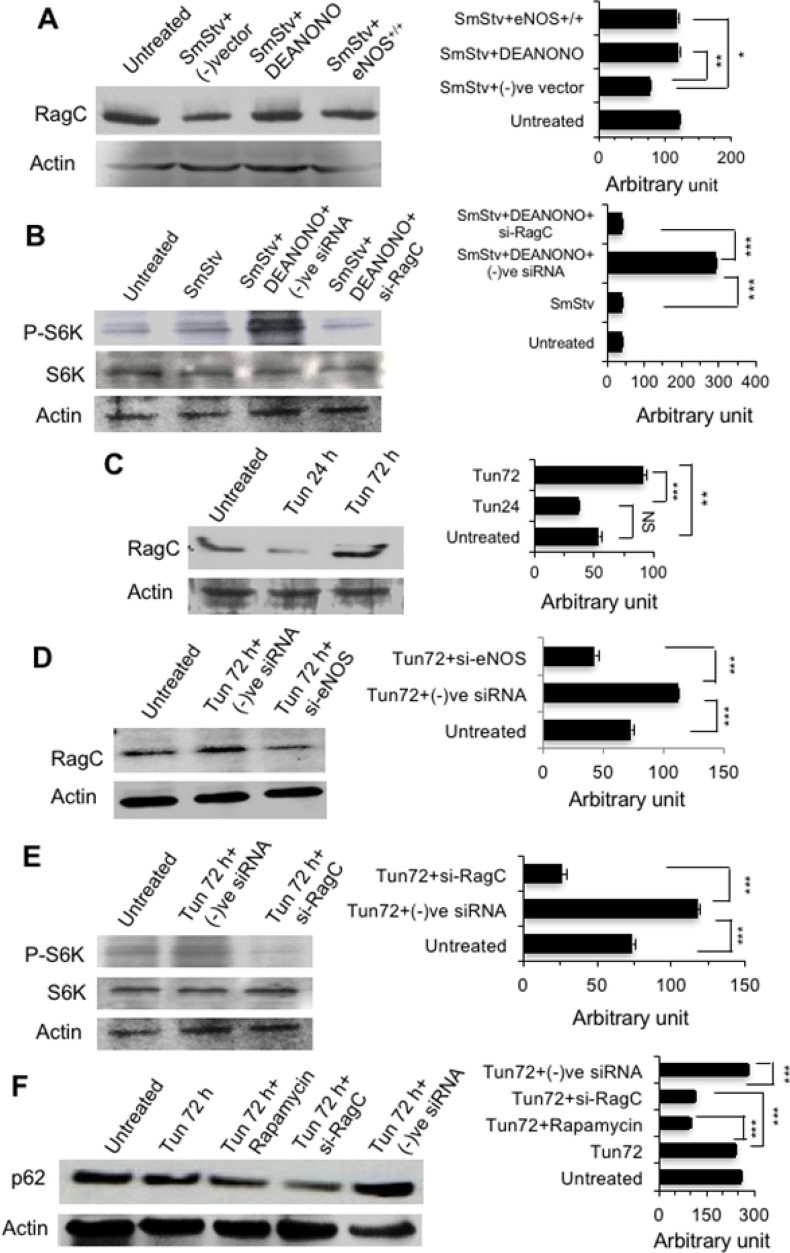
eNOS activates mTORC1 through RagC to inhibit autophagic degradation **(A)** Representative Western blots showing expression of RagC in PC-3 cells. Cells were transfected with eNOS construct (for ectopic expression) or empty vector (for negative control) followed by serum starvation in the presence or absence of NO donor DEANONO for 6 h and expression of RagC was determined on W. blot using anti-RagC antibody. The bar diagram at right shows quantification of RagC from three different experiments as measured by Image J software. **(B)** Representative Western blots showing expression of S6 kinase (S6K) and phosphorylated S6 kinase (P-S6K) in PC-3 cells. Cells were treated with siRNA specific for RagC (si-RagC) or negative control siRNA for 24 h followed by followed by serum starvation in the presence or absence of DEANONO for 6 h and expression of S6K and P-S6K was determined on W. blot using anti-S6K antibody and anti-P-S6K antibody, respectively. The bar diagram at right shows quantification of P-S6K from three different experiments as measured by Image J software. **(C, D)** Representative Western blots showing expression of RagC. Cells were treated with 10 μg/ml Tun for 24-72 h and expression of RagC was determined using anti-RagC antibody. In case of D, cells were treated with siRNA specific for eNOS (si-eNOS) or negative control siRNA for 24 h followed by Tun treatment (10 μg/ml, 72 h). The bar diagrams at right show quantification of RagC from three different experiments as measured by Image J software. **(E)** Representative Western blots showing expression of S6 kinase (S6K) and phosphorylated S6 kinase (P-S6K) in PC-3 cells. Cells were treated with si-RagC or (−)siRNA for 24 h followed by Tun treatment (10 μg/ml, 72 h) and expression of S6K and P-S6K was determined using anti-S6K antibody and anti-P-S6K antibody, respectively. The bar diagram at right shows quantification of P-S6K from three different experiments as measured by Image J software. **(F)**. Representative Western blots showing expression of p62. Cells were treated with rapamycin, si-RagC or (−)siRNA for 24 h followed by Tun treatment (10 μg/ml, 72 h) and expression of p62 was determined using anti-p62 antibody. The bar diagram at right shows quantification of p62 from three different experiments as measured by Image J software. In all cases, actin was used as loading control.

As RagC was found to negatively modulate autophagy [24], we investigated if eNOS could activate mTORC1 through regulation of RagC. First to evaluate the impact of nitric oxide (NO) or eNOS on RagC expression, PC-3 cells were subjected to serum starvation (6 h without FBS) in the presence of DETA-NONOate (NO donor) or ectopically expressed eNOS. Serum starvation reduced RagC expression which was rescued by DETA-NONOate or eNOS over expression (Figure 6A). Next, serum starved PC-3 cells were treated with siRNA against RagC and role in NO dependent mTOR activation was evaluated. NO preserved S6K phosphorylation (a definitive indicator of mTOR activation) which was abrogated by down-regulation of RagC (Figure 6B). Expression of RagC was investigated in Tun treated PC-3 cells and found higher in 72 h compared to 24 h of Tun treatment (Figure 6C). To investigate if eNOS could modulate expression of RagC, cells were treated with siRNA against eNOS and RagC expression was measured. Down-regulation of eNOS substantially reduced RagC expression confirming that Tun induced RagC expression was modulated by eNOS (Figure 6D). Lastly, siRNA against RagC convincingly reduced S6K phosphorylation (Figure 6E) depicting that Tun induced eNOS could modulate RagC expression, which in turn, activated mTOR. To our knowledge, this is the first demonstration that NO donor or eNOS could induce RagC expression followed by modulation of mTOR activation.

Role of mTORC1 and RagC on p62 accumulation was determined in the presence of rapamycin (inhibitor of mTORC1) or after knocking down RagC. Rapamycin or si-RagC reduced p62 accumulation by ∼2 fold compared to Tun treatment alone (Figure 6F). Taken all data together, eNOS activates mTORC1 through eNOS-RagC pathway and inhibits autophagic degradation.

### p62 accumulation facilitates aggregation of ubiquitinylated protein and ROS induced cell death

**Figure 7:**
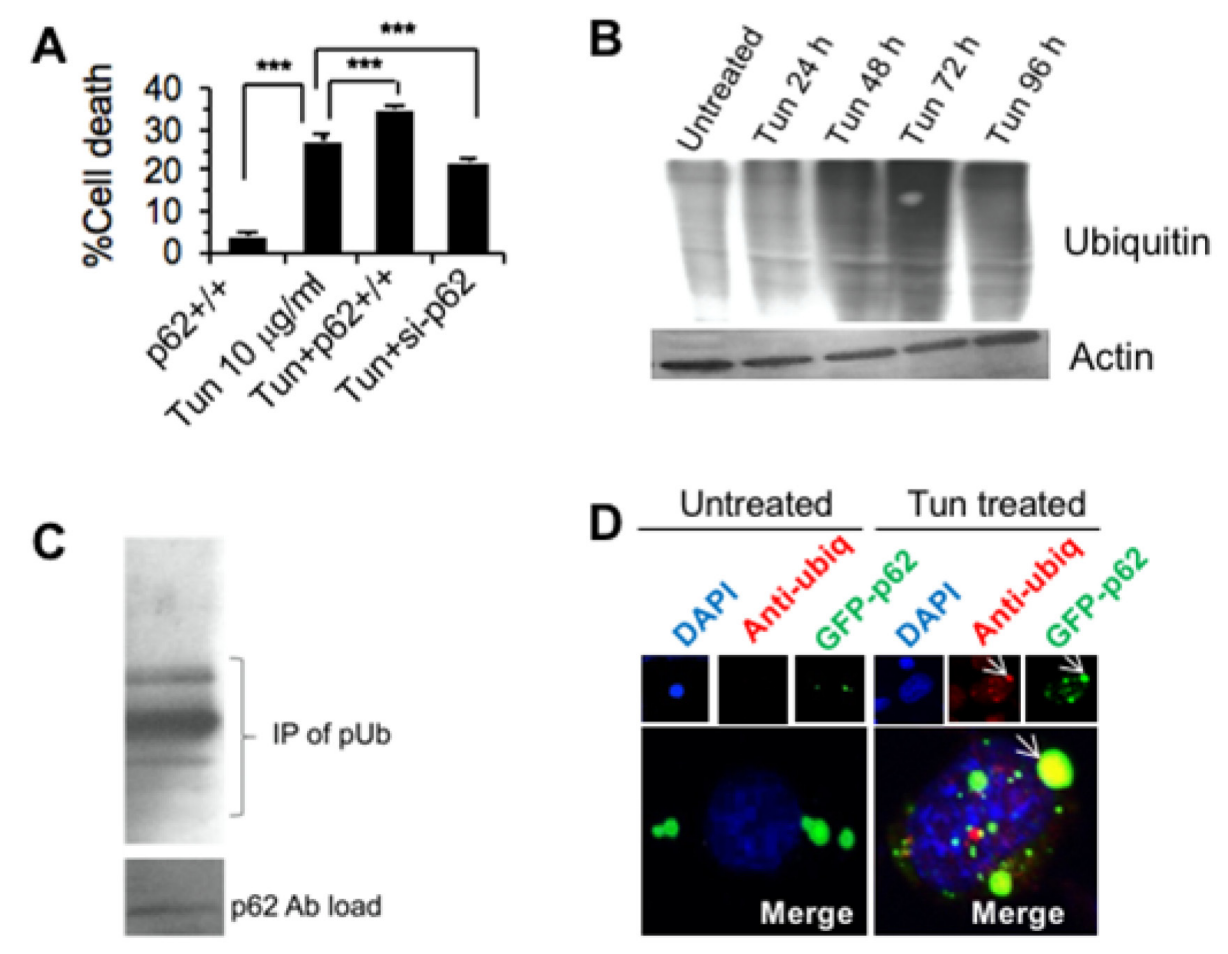
p62 accumulation facilitates aggregation of ubiquitinylated protein and ROS induced cell death **(A)** Effect of p62 down-regulation and up-regulation on PC-3 cell death. The cells were transfected with siRNA specific to p62 (for knockdown) or p62 construct (for ectopic expression) or scrambled siRNA or empty vector for the corresponding negative control followed by treatment with 10 μg/ml of Tun for 72 h and cell viability was measured by WST-1 staining. **(B)** Western blot showing accumulation of ubiquitinylated proteins. PC-3 cells were treated with 10 μg/ml of Tun for 72 h and the cell extracts were subjected to W. blot using anti-ubiquitin antibody. **(C)** Immunoprecipitation of ubiquitinylated proteins with anti-p62 antibody as described in Materials and Methods. Lower panel shows the antibody (p62) load. **(D)** Confocal image showing co-localization of p62 and ubiquitinylated protein in Tun (72 h) treated PC-3 cells (indicated by arrow).

To ascertain the effect of p62 on cell death we knocked down p62 and also over expressed p62 in PC-3 cells along with treatment with Tun (10 μg/ml, 72 h). Interestingly, we found that knockdown of p62 significantly (p<0.01) reduced cell death, but over expression of p62 significantly (p<0.001) increased Tun dependent cell death (Figure 7A). We next investigated how accumulation of p62 stimulated cell death. Our data showed an accumulation of ubiquitinylated proteins in Tun treated cells which markedly increased between 72 and 96 h (Figure 7B). To investigate if p62 could associate with ubiquitinylated proteins, co-immunoprecipitation and confocal imaging were performed. As shown in Figure 7C, p62 antibody precipitated ubiquitinylated proteins indicating an association with p62 and ubiquitinylated proteins. Further, confocal imaging showed a direct association of p62 with ubiquitinylated proteins (Figure 7D, merge picture). As shown in Figure 3A-3D, intracellular aggregation of misfolded proteins promoted ROS generation leading to cell death [25]. Taken together, data suggest that p62 accumulation resulted in the cellular deposition of misfolded of toxic protein aggregates leading to the ROS generation. Sustained accumulation of ROS destabilized mitochondrial membrane potential and triggered mitochondrion-dependent apoptosis.

### eNOS plays a role in DNA fragmentation or late stage apoptosis

**Figure 8:**
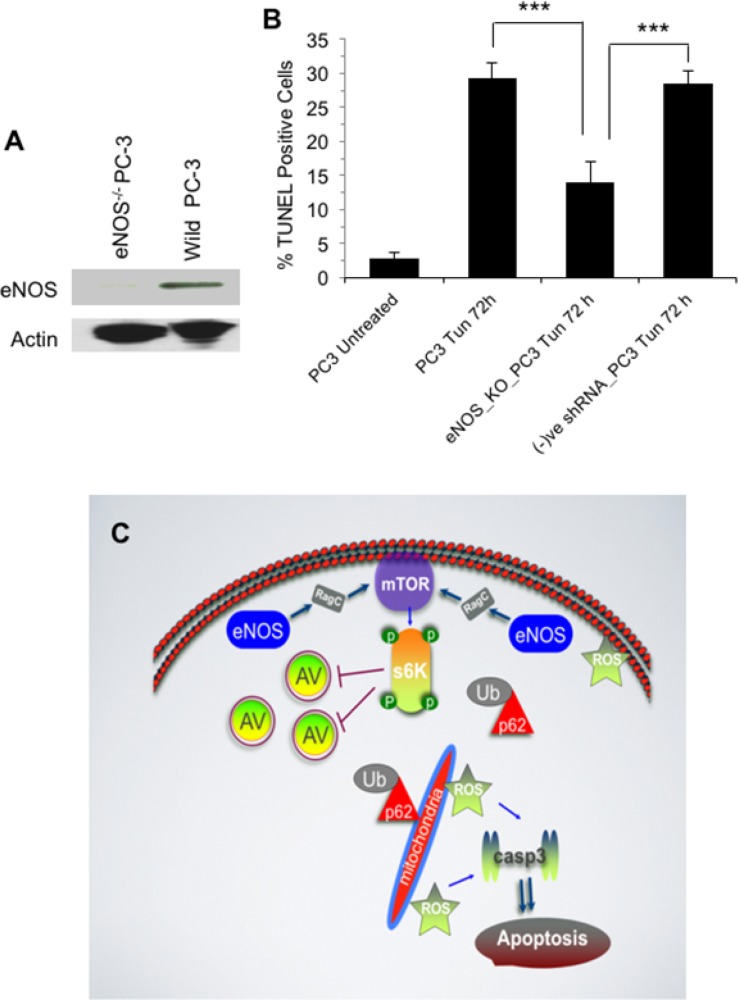
eNOS plays a role in DNA fragmentation or late stage apoptosis **(A)** Validation of eNOS knockout in PC-3 cells by Western blotting of eNOS. PC-3 cells were stably transfected with lentiviral shRNA targeted to eNOS to prepare eNOS knockout (eNOS^−/−^) PC-3 cells or a vector containing scrambled shRNA for a negative control. Cell extracts (both wild type and transfected) were subjected to W. blot using anti-eNOS antibody. **(B)** TUNEL Assay. Both wild type and transfected PC-3 cells were treated with Tun (10 μg/ml, 72 h) and TUNEL assay was performed on a flow cytometer. **(C)** Schematic representation showing ER stress induced eNOS signaling. Sustained ER stress induces eNOS and through RagC activates mTORC1, which then negatively modulates autophagic process through phosphorylation of S6 kinase. Inhibition of autophagy results into accumulation of p62, which positively influences aggregation of ubiquitinated proteins and compromises clearance of misfolded toxic protein aggregates. The p62-ubiquitinated protein aggregates cause mitochondrial ROS generation and caspase 3-dependent apoptosis.

Role of eNOS in late stage apoptosis was confirmed by TUNEL assay of eNOS knockout PC-3 cells in the presence or Tun. To prepare a stable eNOS^−/−^PC-3 cells, cells were transfected with lentiviral shRNA targeting eNOS. Lack of eNOS expression in eNOS^−/−^PC-3 cells was first confirmed (Figure 8A). Negative shRNA control transfected cells showed eNOS expression (data not shown). On TUNEL assay, Tun-treated eNOS^−/−^ cells showed almost 58% reduction (after subtracting background) (p<0.001) of apoptosis compared to Tun-treated wild type PC-3 cells (Figure 8B). Negative shRNA control transfected PC-3 cells showed similar results as the wild type PC-3 cells (data not shown).

Taken all data (described above) together, sustained Tun-induced ER stress up-regulates eNOS, which in turn, activates mTORC1 (as measured by phosphorylation of S6 kinase) through RagC. This results into an accumulation of p62 which facilitates aggregation of ubiquitinated protein thus compromising clearance of misfolded toxic protein aggregates. Lastly, association of p62 proteins and misfolded proteins promote ROS mediated mitochondrial apoptosis (Figure 8C).

## DISCUSSION

Cell’s decision to live or die under stress conditions is determined by the crosstalk between signaling pathways that regulate these processes. Cells make mutually exclusive decisions (‘live’ or ‘die’) by employing pleiotropic pathways or proteins. It is largely unknown how cells succeed to switch on or off robustly specific stress response using an integrated network. From whole genome expression microarray analysis of Tun-treated PC-3 cells at two time points, we show that under sustained ER-stress, up-regulation of eNOS triggers death switch.

An increase in misfolded proteins due to change in ER folding capacity is monitored and resolved by three genes (IRE1, PERK and ATF6) of unfolded protein response (UPR) [3, 6, 7]. These genes not only restore ER homeostasis but also can trigger apoptosis under sustained ER stress or if the stress cannot be resolved. Studies suggest that both autophagy and apoptosis are controlled by UPR. Particularly, UPR activates autophagy as a control strategy to delay apoptosis since it also serves as an activator of apoptosis [9]. Further, UPR is shown to activate mTOR pathway, which in turn promotes apoptosis, while inhibits autophagy [26]. However, until now nothing was known how mTOR gets activated by the UPR. Here, we demonstrated that mTORC1 could be activated by eNOS (Figure 5G).

Autophagy induced by stress or starvation involves the sequestration of cellular organelles or infectious pathogens by double-layered membranes called autophagosomes, which ultimately fuse with lysosomes and their contents are degraded [10]. Such self-digestion sustains cell survival during stress and eliminates damaged proteins and organelles that accumulate during stress. Autophagy involves 3 distinct stages: vesicle nucleation (formation of autophagophore around organelles), elongation and completion, fusion of the double-membraned autophagosome with the lysosome to form an autolysosome, and lysis of the autophagosome inner membrane and breakdown of its contents inside the autolysosome. This process occurs at a basal level and is regulated by multiple different signaling pathways. The formation of the autophagophore involves at least 16 autophagy-related proteins [10]. Two ubiquitin-like conjugation systems are involved in autophagy. These systems produce modified complexes of autophagy regulators (Atg8-PE and Atg5-Atg12-Atg16) that may determine the formation and size of the autophagosome. Nucleation, expansion, uncoating, and completion of the autophagosome then occur, priming it to fuse with lysosomes [27]. The initiating signal for autophagosome formation is poorly understood, but the mTOR is a negative regulator, and the extent of autophagy is regulated by proteins upstream of mTOR signaling, including PTEN, PDK1, Akt, and TSC1/2 [28] as well as downstream targets such as elongation factor-2 kinase [29] and S6 kinase [28]. Here, we have shown that two upstream proteins of mTOR, eNOS and RagC are involved in the activation of mTOR, which, in turn negatively regulates autophagy (Figure 6).

The relationship between autophagy and apoptosis is complex as autophagy allows cell to escape stress and avoids or delays death response, whereas in some cases, autophagy is followed by apoptosis [30]. As autophagy and apoptosis are related with several disease conditions, from neuronal degenerative disease to cancer [4, 5], it is important to identify the subtle link between these two. Here we found that inhibition of autophagy is the first step for stimulation of apoptosis. In our whole genome expression microarray of the autophagy-to-apoptosis juncture, a few genes were markedly up regulated- of which eNOS expression was the highest. We found that during sustained ER stress expression of eNOS stimulated activation of mTORC1 that negatively modulated autophagy. mTORC1 could inhibit autophagosome formation by associating with the ULK1-ATG13-FIP200 complex and phosphorylating ULK1 and Atg13 [21]. In models of Huntington’s disease Sarkar et al. [31] recently found that eNOS expression could negatively modulate autophagy.

How does inhibition of autophagy stimulate apoptosis? We found that intracellular accumulation of SQSTM1/p62 could sensitize cell for apoptosis when autophagy is inhibited. In general, p62 binds with the ubiquitinylated protein aggregates followed by their autophagic degradation [32]. Accumulation of p62 not only compromises ubiquitin/proteasome system [23], but also influences accrual of unfolded toxic protein aggregates in ER and mitochondria. Since p62 along with polyubiquitinylated proteins gets also degraded during the process of autophagic degradation, up-regulation of p62 at the transcriptional level can be compensated when autophagy is activated upon cellular stress. However, prolonged ER stress stimulated eNOS expression, which in turn, suppressed autophagic system through RagC-mTORC1 pathway resulting in cellular accumulation of p62 and unfolded toxic protein aggregates. RagC, a Rag GTPase family of protein could activate mTOR signaling in the presence of amino acid and thus could negatively modulate autophagy [33]. It has already been shown that tuberous sclerosis complex (TSC) could activate mTOR in the presence of NO resulting into inhibition of autophagy [24]. However, TSC independent mTOR activation could be achieved through Rag GTPase. Here, for the first time we showed that NO donor or eNOS could induce RagC expression followed by modulation of mTOR activation. Consistent with Mathew et al. [32], we also found that p62 accumulation impaired redox regulation followed by ROS generation. ROS generation was due to an accumulation of p62 and unfolded toxic protein aggregates in the ER and mitochondria that impaired functional homeostasis. Substantial levels of ROS could alter mitochondrial membrane potential and caspase 3 activation and also could trigger ER mediated apoptotic signal.

Sustained ER stress could trigger both autophagy and apoptosis and at the autophagy-to-apoptosis juncture, upregulation of eNOS negatively modulated autophagy through activation of mTORC1. Subsequently, inhibition of autophagy influenced p62 and ROS accumulation in the cells and triggered apoptotic signal. One could argue that there would be more cell death, if the autophagic process is denied with an autophagic blocker while cells are under stress. In our preliminary experiment (Figure 2F), a synergistic cell death of PC-3 occurred when treated with a mixture of chloroquine (autophagy blocker) and tunicamycin suggesting that the initial autophagic activation was important for cell survival. So, it is possible that a combination of chloroquine and tunicamycin might be useful therapeutics for cancer.

## MATERIALS AND METHODS

### Materials

Tunicamycin, chloroquine, N-acetyl cysteine (NAC), DETA-NONOate, mammalian cell lysis kit, Rabbit anti-actin antibody, DMEM:F12 medium, Mission esiRNA for SQSTM1, and eNOS siRNA were purchased from Sigma-Aldrich. Glycan Differentiation kit, WST-1 cell viability kit, and X-tremeGENE HP DNA Transfection reagent were obtained from Roche. ROS detection kit, gold antifade mounting medium, NuPAGE Novex 4-12% Bis-Tris gels, goat anti-rabbit IgG-alexa 488, Goat-anti-rabbit IgG-Alexa Fluor 568, First Strand cDNA synthesis kit, Fetal Bovine Serum were from Invitrogen. JC1 mitochondrial membrane potential assay kit was from Cayman. Apodirect TUNEL assay kit was purchased from Millipore. Ultralow attachment tissue culture plate was from Nunc. TurboFect siRNA Transfection Reagent, DAPI, and HRP chemiluminescence substrate were from Thermo Scientific. Anti-ubiquitin antibody (clone P4D1) was from Biolegend. Anti rabbit antibodies against LC3, p62, and calnexin, Rabbit mAb phosphor-p70 S6 Kinase (Thr 389), mouse anti-eNOS mAb were obtained from Cell signaling Technology. pcDNA3-eNOS-GFP was obtained from Addgene. LC3-GFP and p62-EGFP constructs were kind gifts from Dr. Beth Levine (Columbia University, NY) and Dr. Terje Johansen (University of Tromso, Norway), respectively.

### Cell culture

Androgen-independent metastatic prostate cancer cell line PC3 (ATCC, Manassas, VA) were cultured in DMEM:F12 (1:1) supplemented with 10% fetal bovine serum (FBS) (Quality Biologicals, Gaithersburg, MD), 100 units/ml penicillin G sodium and 100 μg/ml streptomycin sulfate (Sigma) in the presence of 5% CO_2_ at 37°C. Unless otherwise stated, the following dose was used for treatment of cells with chloroquine (50 μg/ml) [34] and NAC (2.5 mM) [35]. Serum starvation of cells was performed without FBS for 6 h.

### Cell viability and cell apoptosis

Cell viability was assessed on 96-well plate using WST-1 colorimetric assay according to the manufacturer’s instructions [36] (Roche). TUNEL assay using Apodirect TUNEL assay kit (Millipore) for cell apoptosis was performed on a flow cytometer (FACSCanto, Becton Dickinson) using channel FL1. Also, to analyze whether the cell death was apoptosis the cells (5 × 10^6^) were incubated with cleaved caspase 3 antibody (cell signaling Technology) at a ratio of 1: 100 for 1 h at 37°C followed by secondary goat anti-rabbit alexa 488 antibody (Invitrogen) (1: 1000) for 30 min at 37°C. Finally, the samples were washed 3 times followed by flow cytometry at FL1 channel.

### LC3-GFP punctate staining

PC-3 cells stably expressing LC3-GFP were plated onto 2 well chamber slides (BD) and subjected to treatment with tunicamycin (10 μg/ml) for 24, 48, 72, and 96 h. Cells were fixed in 3.7% formaldehyde (Sigma), examined by confocal microscopy (Olympus FV-1000) and digital images were obtained with CCD camera of the microscope software. More than 5 puncta per cell was considered autophagy. Cells were counted under in each field and 5 different fields were scored for statistical analysis.

### Analytical procedures

Protein concentration was measured by Bradford assay using bovine serum albumin (BSA) as a standard as previously described [37]. Analytical polyacrylamide gel electrophoresis (PAGE) in the presence of SDS (2%) and β-mercaptoethanol was carried out on a fixed percentage of acrylamide gels (12% or 15%) as reported elsewhere [36]. Occasionally, NuPAGE Novex 4-12% Bis-Tris gels (Invitrogen) under reducing condition as performed. For protein detection on membranes, polyacrylamide gels were subjected to dry Western blot transfer (Invitrogen) and the nitrocellulose membranes were incubated with respective primary antibodies [38]. After washing, the bound antibody was captured by secondary antibody-horseradish peroxidase (HRP) conjugate followed by development with HRP chemiluminescence substrate (Thermo Scientific). The light signal corresponding to the protein band was captured on X-ray film (Kodak) and quantitated by Image J software. For each protein expression, at least three Western blots were performed independently. The presence of N- and O-glycosylation in tunicamycin treated PC-3 cells was investigated using glycan differentiation kit (Roche, Indianapolis, IN) [39]. Antibodies for Western blotting were used as the following dilutions: Anti rabbit LC3 (1:1000), anti rabbit p62 (1:1000), anti rabbit Calnexin (1:1000), monoclonal anti rabbit phosphor-p70 S6 Kinase (Thr 389), monoclonal anti mouse eNOS (1:1000), anti rabbit beta actin (1:1000), anti rabbit ubiquitin (1:1000) (clone P4D1). Secondary antibodies (anti rabbit IgG and anti mouse IgG) from KPL (Gaithersburg, MD) were used in 5000-fold dilutions.

### Immunoprecipitation

PC-3 cells (6 wells of 5x 10^4^ cells/well were used) were lysed with 1 ml of immunoprecipitation buffer (0.05 m Tris-HCl, pH 7.4, 0.15 m NaCl, 0.25% deoxycholic acid, 1% Nonidet P-40, 1 mm EDTA) supplemented with 1% protease mixture inhibitor (Sigma), centrifuged for 10 min at 13,000 × *g* and the supernatant collected. The supernatant (200 μl) was mixed with rabbit anti-p62 antibodies at the (1:50) concentration and incubated at 4°C on a rocker platform overnight. Two hundred microliter of goat anti-rabbit IgG-magnetic beads were then added to the mix and continued incubation for another hour at room temperature. The antigen-antibody complex was then separated using a magnetic stand and the supernatant discarded. The beads were washed three times with 300 μl of lysis buffer and were then resuspended in 30 μl of electrophoresis sample buffer and heated to 95°C for 10 min. The beads were placed on the magnetic stand, the supernatant collected and used for Western blot analysis.

### In-gel digestion for LC-MS/MS protein identification

Coomassie-stained protein bands were excised, cut into approximately 1 × 1 mm pieces and dehydrated with methanol for 5 min. The gel pieces were then washed as follows: 1 × 5 min with 30% methanol/70% water, 2 × 10 min with water, and 3 × 10 min with 100 mM ammonium bicarbonate (NH_4_HCO_3_)/30% acetonitrile. Gel pieces were dried in a SpeedVac. Protein disulfide bonds were reduced with 10 mM tris(hydroxypropyl)phosphine (TCEP) in 100 mM NH_4_HCO_3_ for 60 min at 56°C, followed by alkylation with 55 mM iodoacetamide in 100 mM NH_4_HCO_3_ for 45 min at room temperature in the dark. The gel pieces were washed with 100 mM NH_4_HCO_3_ for 15 min and dehydrated with acetonitrile followed by complete drying in a SpeedVac. Gel pieces were rehydrated in trypsin solution (15 ng/μL trypsin in 50 mM NH_4_HCO_3_) on ice for 45 min. Excess trypsin solution was discarded, replaced with 50 mM NH_4_HCO_3_ and incubated overnight at 37°C. Digestion buffer was collected and saved. Peptides were extracted once with 50 mM NH_4_HCO_3_, once with acetonitrile and twice with 5% formic acid in 50% acetonitrile; each extraction was performed by incubating at 37°C for 15 min with vortexing. All supernatants were combined, dried in a SpeedVac and de-salted using PepClean C18 Spin columns (Pierce). For LC-MS/MS analysis, de-salted peptides were separated by nanoscale reverse-phase liquid chromatography using an Xtreme Simple nanoLC system (CVC/Micro-Tech). The analytical column was prepared by packing 1.7μm 200Å C18 resin (Prospereon Life Sciences) into a laser-pulled fused silica capillary (75 μm inner diameter, 10.5 cm length, 10μm tip; Sutter Instruments) using a pressure injection cell (Next Advance). Peptides were injected into the sample loop using an Endurance autosampler (Spark Holland, Brick, NJ) and were loaded onto the column with 95 % solvent A (0.5% acetic acid in water). A 40 min LC gradient method from 5 – 35% B (80% acetonitrile, 0.5% acetic acid) with a post-split flow rate of 0.3 μl/min was used to elute the peptides into the mass spectrometer. The LTQ-Orbitrap mass spectrometer (Thermo Electron) was equipped with a nanospray ionization source. The spray voltage was 1.5 kV and the heated capillary temperature was 180°C. MS1 data were acquired in the profile mode in the Orbitrap with a resolution of 60,000 at 400 m/z and the top ten most intense ions in each MS1 scan were selected for collision induced dissociation in the linear ion trap. Dynamic exclusion was enabled with repeat count 1, repeat duration 30 sec, and exclusion duration 180 sec. Other mass spectrometry data generation parameters were as follows: collision energy 35%, ion selection threshold for MS/MS 500 counts, isolation width 3 m/z, default charge state 3, and charge state screening enabled. For peptide and protein identification, MS/MS data were acquired using a top 5 data-dependent acquisition method with dynamic exclusion enabled. MS/MS spectra were searched against a UniProtKB human protein database (version Oct 5, 2010; 20,259 reviewed sequences; 75,498 non-reviewed sequences) using **Sorcerer™-SEQUEST®** (Sage-N Research). The quality of peptide and protein assignments was assessed using PeptideProphet and ProteinProphet. Proteins with probabilities of ≥ 1.0 and ≥ 2 unique peptides were accepted as confidently identified peptides.

### Whole genome expression microarray

To delineate the role of candidate genes in the apoptotic process under ER stress and to search for new therapeutic strategies to overcome the resistance of hormone-refractory prostate cancer cells, we performed whole genome expression microarray analysis in Tun-treated PC-3 cells. DNA microarray analysis was performed using the Human v5 Whole Genome OneArray® (Phalanx Biotech, Belmont, CA). RNA quality and integrity were determined utilizing an Agilent 2100 Bioanalyzer (Agilent Technologies, Palo Alto, CA) and a NanoDrop spectrophotometer (Thermo Scientific, Wilmington, DE). Only high quality RNA, having a RIN of >7.0, and absorbance ratios A260/A280 >1.8 and A260/A230 >1.6, was utilized for further experimentation. RNA was converted to double-stranded cDNA and amplified using *in vitro* transcription that included amino-allyl UTP, and the RNA product was subsequently conjugated with with Cy5™ NHS ester (GEH Lifesciences). Fragmented RNA was hybridized at 42C overnight using the HybBag mixing system with 1X OneArray® Hybridization Buffer (Phalanx Biotech), 0.01 mg/ml sheared salmon sperm DNA (Promega, Madison, WI), at a concentration of 0.025 mg/ml labeled target. After hybridization, the arrays were washed according to the OneArray® protocol.

Raw intensity signals for each microarray were captured using a Molecular Dynamics™ Axon 4100A scanner, measured using GenePixPro™ Software, and stored in GPR format. The data from all microarrays in each experimental set was then passed to Rosetta Resolver (Rosetta Biosoftware) for analysis. Testing was performed by combining technical replicates and performing statistical analyses using Rosetta Resolver’s proprietary modeling techniques.

### Quantitative real-time PCR (qRT-PCR)

For this purpose, total RNA was isolated using TRIzol reagent (Invitrogen) according to the manufac-turer’s protocol. One microgram of total RNA was reverse-transcribed using Cloned AMV First-Strand cDNA synthesis kit (Invitrogen) in a 20-μl total reaction volume, followed by quantitative PCR on ABI 7500 Fast Thermocycler with RT^2^SYBR Green ROX Fast Master Mix (Qiagen) using gene specific primers (see Table 1 for primer sequences). For each exponential amplification, PCR was performed for 1 cycle at 95°C followed by 40 cycles at 95°C and 60°C. To verify amplification specificity, melting curves of the PCR products of each primer set were analyzed. Each experiment was prepared in triplicate, and data are represented as means ± S.D. of at least three independent experiments. To normalize for sample variation, expression of glyceraldehyde-3-phosphate dehydrogenase was determined as an internal control.

### Transfections with expression plasmids and siRNAs

For transient expression, cells were transfected with the following plasmids using X-tremeGENE HP DNA Transfection reagent (Roche): pcDNA3-eNOS-GFP for eNOS expression, p62-EGFP for p62 expression, and LC3-GFP for LC3 expression. To knockdown SQSTM1/p62, and eNOS, corresponding siRNAs were procured from Sigma as follows: eNOS siRNA (Cat No. SASI_Hs01_00174420), and SQSTM1/p62 Mission siRNA (Cat No. SASI_HS01_00118616). For knockdown, PC-3 cells were cultured in 6-well plates and transiently transfected with 25 nM siRNAs using TurboFect siRNA Transfection Reagent (Thermo Scientific). For negative control experiments, non-targetable negative control siRNA (Dharmacon) or the corresponding empty vector was used.

### Analysis of reactive oxygen species (ROS) generation and change of mitochondrial membrane potential

ROS was analyzed using the ROS detection kit from (Invitrogen) according to the manufacturer’s instructions and quantitated on a spectrofluorimeter (SpectraMax M5 Multimode Microplate Reader from Molecular Devices, CA). Mitochondrial membrane potential change was analyzed using JC-1 (Cayman) according to the manufacturer’s instructions on a flow cytometer as well as on a fluorescence plate reader. Data are represented as the amount of J-aggregate signified by the amount of red fluorescence or the ratio of J- aggregate (Em 595): J-monomer (Em 538).

### Statistical analysis

The statistical analyses were performed using one-way analysis of variance (ANOVA) followed by Turkey-Kramer multiple comparisons (Graphpad Instat, version 3). The differences were considered significant when p<0.05. Unless otherwise stated, all data are representative of three independent experiments with S.D. indicated by error bars. P values are determined by two-tailed student t-test and <0.05, <0.01 and <0.001 are indicated by one, two and three asterisks, respectively.

## Abbreviations

ER: endoplasmic reticulum
ROS: reactive oxygen species
Tun: tunicamycin
CQ: chloroquine
PBS: phosphate-buffered saline (10 mM phosphate, 140 mM NaCl, pH 7.5)
